# Supplementary material for: Effect of hydrophobic cations on the oxygen reduction reaction on single‒crystal platinum electrodes
Source: Nat Commun. 2018 Nov 5;9:4378. doi: 10.1038/s41467-018-06917-4 (PMC6218472; doi:10.1038/s41467-018-06917-4)
Supplement: Supplementary file 1 — Supplementary Information [file 41467_2018_6917_MOESM1_ESM.pdf]

Supplementary information

Effect of hydrophobic cations on the oxygen reduction reaction on single-crystal platinum electrodes

Tomoaki Kumeda[1], Hiroo Tajiri[2], Osami Sakata[3], Nagahiro Hoshi[1] and Masashi Nakamura[1]*

[1]Department of Applied Chemistry and Biotechnology, Graduate School of Engineering, Chiba University, Chiba, Japan. [2]Research and Utilization Division, Japan Synchrotron Radiation Research Institute / SPring–8, Hyogo, Japan. [3]Synchrotoron X-ray Station at SPring-8, National Institute for Materials Science, Hyogo, Japan.

*e-mail: mnakamura@faculty.chiba-u.jp

Contents:

Supplementary Figures

Supplementary Table

Supplementary Figures

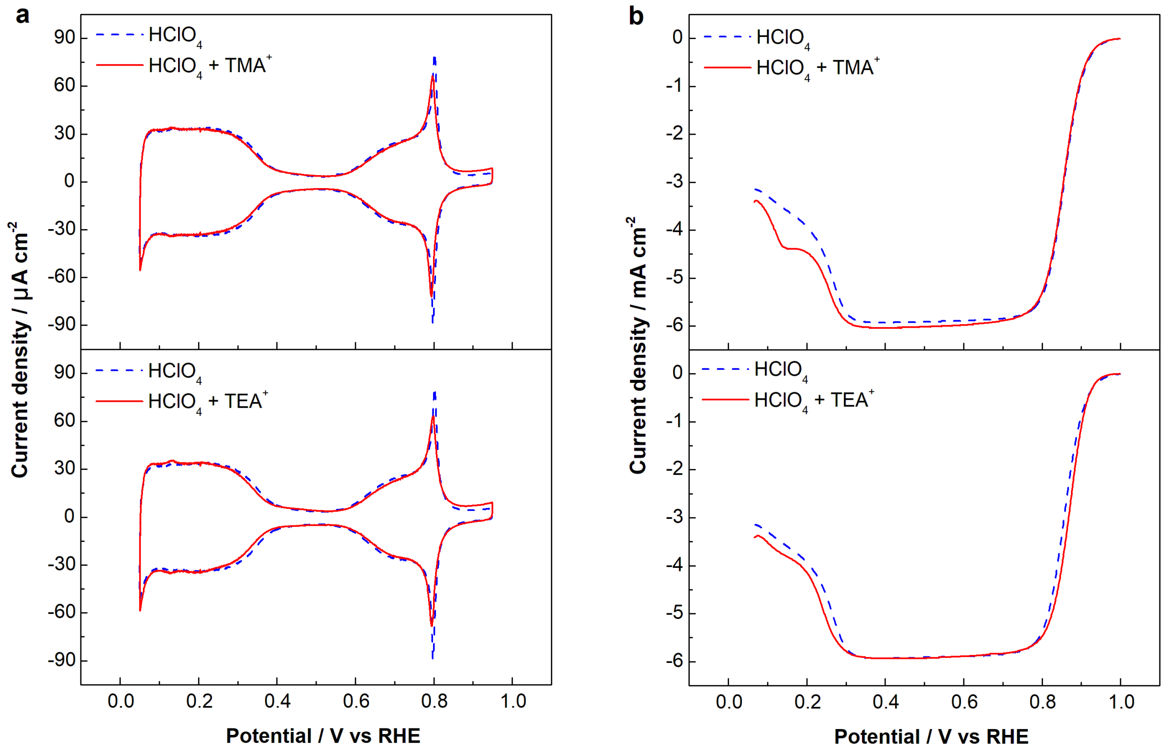

Supplementary Figure 1 | a, CVs and **b,** ORR voltammograms of Pt(111) in 0.1 M $HClO_4$ containing 10^{-3} M TMA^+ and 10^{-3} M TEA^+. CVs were obtained in the solutions saturated with Ar. The scanning rate is 0.050 V s^{-1}. ORR voltammograms were obtained in the solutions saturated with O_2 and the potential was scanned from 0.05 V in the positive direction. The scanning rate is 0.010 V s^{-1} and the rotation rate of the electrode is 1600 rpm.

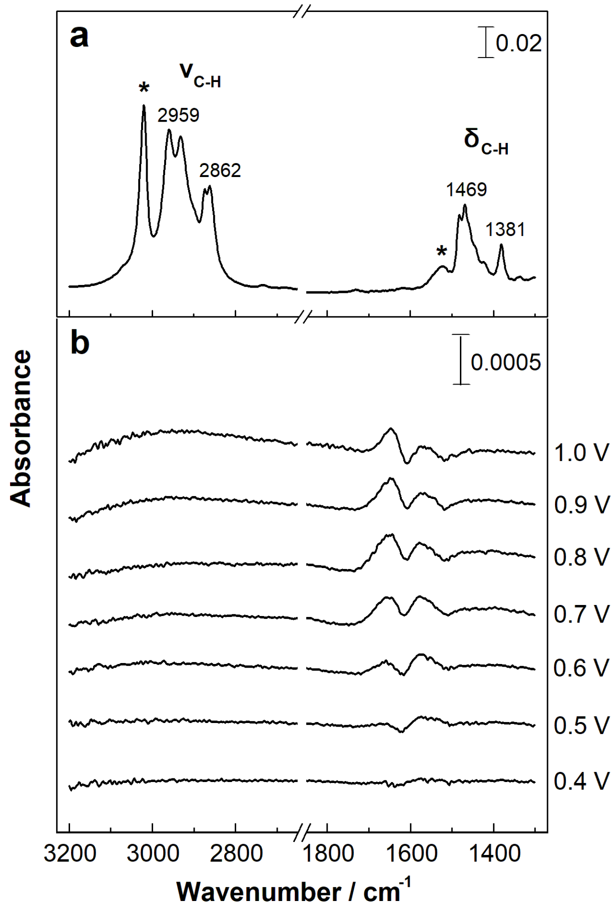

Supplementary Figure 2 | a, Transmission IR spectra of $THAClO_4$ in chloroform. The IR bands around 2900 and 1400 cm^{-1} are attributed to the CH stretching and the CH bending modes of THA^+, respectively. The IR bands marked with an asterisk are derived from bulk chloroform. **b,** Potential dependence of IR spectra on Pt(111) modified with THA^+ in 0.1 M $HClO_4$. The potential of the background spectra is 0.30 V vs RHE.

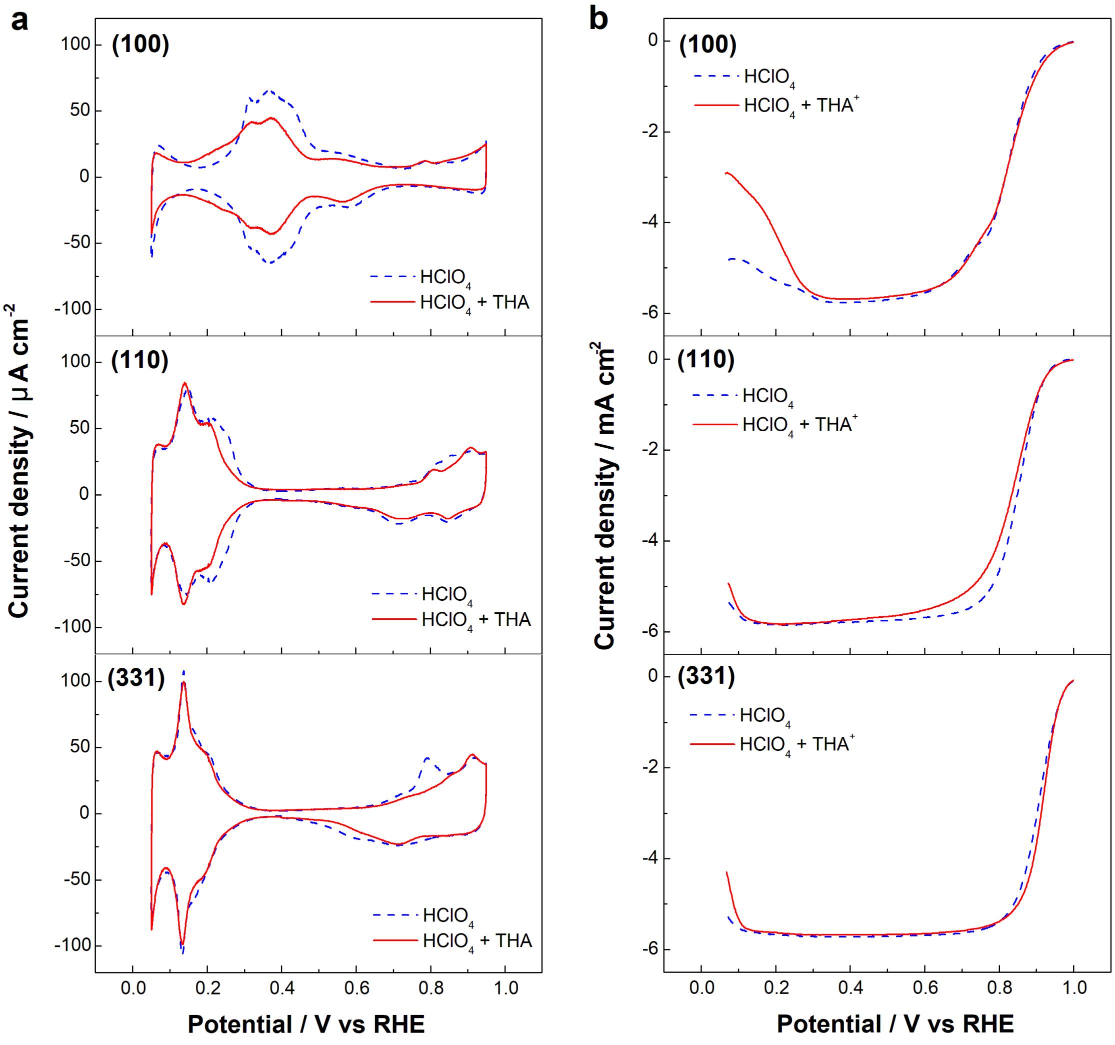

Supplementary Figure 3 | a, CVs and **b,** ORR voltammograms of Pt(100), Pt(110), and Pt(331) = 3(111)−(111) in 0.1 M $HClO_4$ containing 10^{-6} M THA^+. CVs were obtained in the solutions saturated with Ar. The scanning rate is 0.050 V s^{-1}. ORR voltammograms were obtained in the solutions saturated with O_2 and the potential was scanned from 0.05 V in the positive direction. The scanning rate is 0.010 V s^{-1} and the rotation rate of the electrode is 1600 rpm.

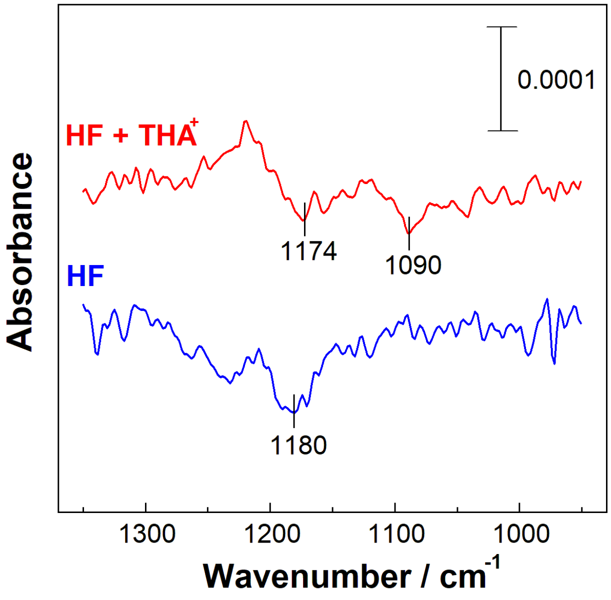

Supplementary Figure 4 | IR spectra of adsorbed D_2O on Pt(111) and Pt(111) modified with THA^+ at 0.90 V vs RHE. The bands at 1180 and 1090 cm^{-1} are assigned to the DOD bending mode of adsorbed hydrogen bonded D_2O and adsorbed monomer, respectively. The potential of the background spectra is 0.30 V vs RHE.

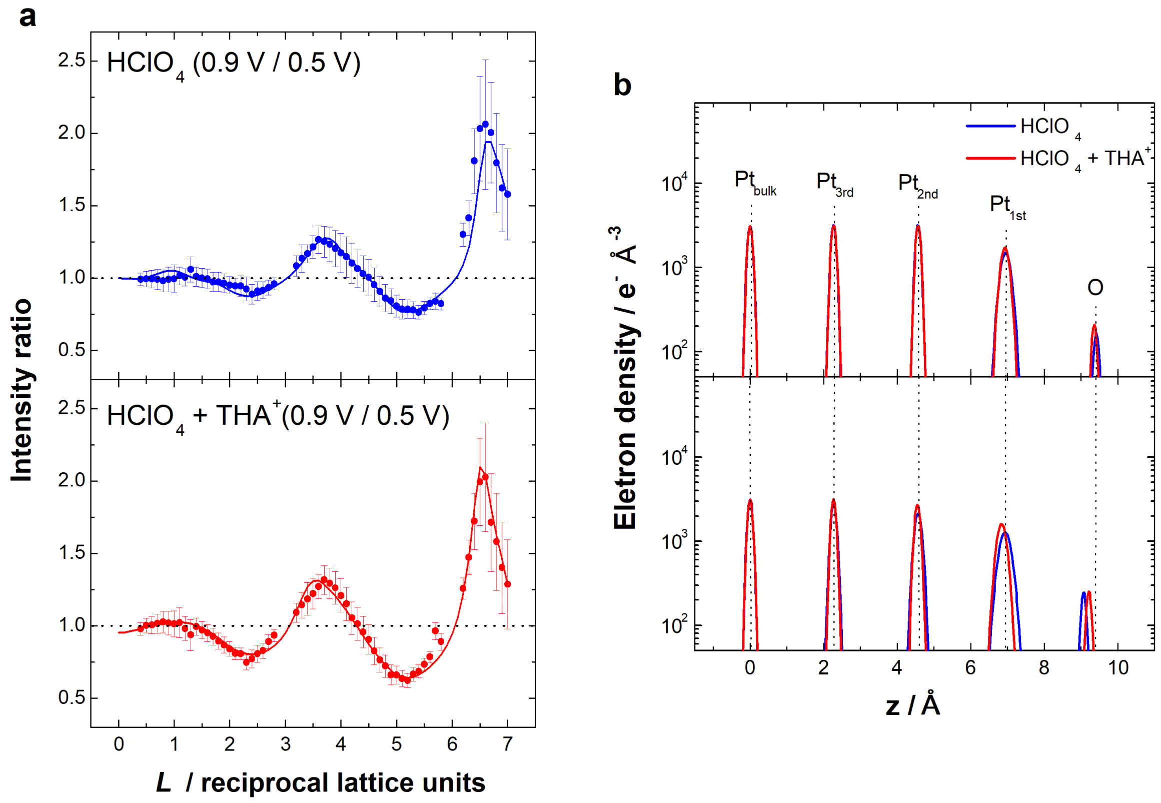

Supplementary Figure 5 | a, Specular CTR profiles at 0.90 V normalized to the data at 0.50 V. **b**, The electron density profiles of the optimized model at 0.50 V and 0.90 V.

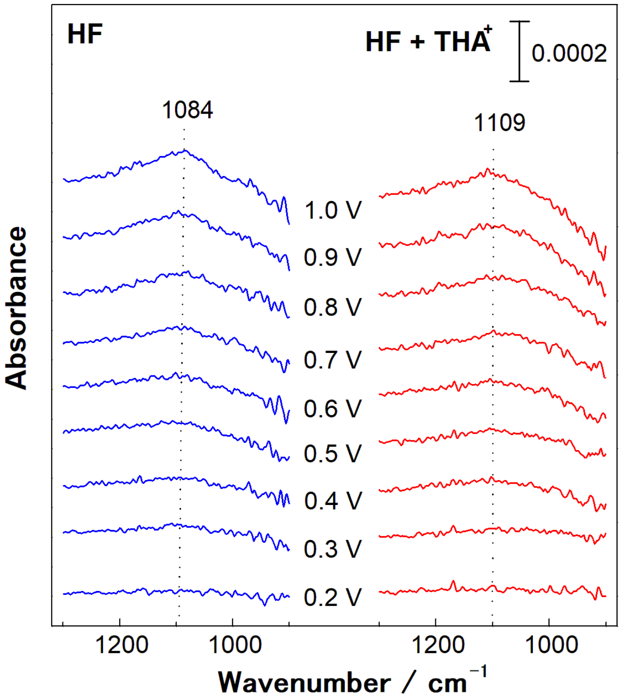

Supplementary Figure 6 | Potential dependence of IR spectra of adsorbed OH on Pt(100) and Pt(100) modified with THA^+ in 0.1 M HF saturated with Ar. Since the OH_{ad} occur on Pt(100) above 0.3 V vs RHE[14], the potential of the background spectra is selected at 0.10 V vs RHE.

Supplementary Table I Structural parameters of the Pt(111) in 0.1 M $HClO_4$ with and without 10^{-6} M THA^+ at 0.5 V and 0.9 V vs RHE.

	**0.50 V**		**0.90 V**	
	**$HClO_4$**	**$HClO_4$ + THA^+**	**$HClO_4$**	**$HClO_4$ + THA^+**
θ_{O}	0.53 ± 0.11	0.65 ± 0.13	0.78 ± 0.15	0.79 ± 0.15
θ_{Pt1st}	1.00 ± 0.03	1.00 ± 0.03	1.00 ± 0.02	1.01 ± 0.03
θ_{Pt2nd}	1.03 ± 0.02	1.01 ± 0.02	1.03 ± 0.01	1.00 ± 0.02
θ_{Pt3rd}	1.03 ± 0.02	1.02 ± 0.02	1.03 ± 0.01	1.01 ± 0.02
$d_{O-Pt1st}$ / Å	2.47 ± 0.20	2.43 ± 0.14	2.14 ± 0.20	2.38 ± 0.14
$d_{Pt1st-Pt2nd}$ / Å	2.38 ± 0.02	2.36 ± 0.01	2.37 ± 0.02	2.29 ± 0.01
$d_{Pt2nd-Pt3rd}$ / Å	2.30 ± 0.01	2.30 ± 0.01	2.29 ± 0.01	2.28 ± 0.01
